# A high rate of mortality in liver cirrhosis patients after emergency abdominal surgery

**DOI:** 10.1007/s00068-025-02787-w

**Published:** 2025-02-21

**Authors:** Anders Peter Skovsen, Thomas Korgaard Jensen, Ismail Gögenur, Mai-Britt Tolstrup

**Affiliations:** 1 Department of Surgery, Copenhagen University Hospital North Zealand, Dyrehavevej 29, Hillerød, 3400 Denmark; 2 Department of Surgery, Copenhagen University Hospital Herlev, Herlev Ringvej 75, Herlev, 2730 Denmark; 3 Department of Surgery, Center for Surgical Science, Zealand University Hospital, Lykkebaekvej 1, Koege, 4600 Denmark

**Keywords:** Liver cirrhosis, Emergency surgery, Mortality, Morbidity, Postoperative complications

## Abstract

**Purpose:**

In the elective setting, there are high mortality rates for patients with liver cirrhosis after surgery. Few studies focus on emergency surgery. This study investigates mortality and morbidity of patients with cirrhosis undergoing emergency abdominal surgery.

**Methods:**

In a database established at two Copenhagen University Hospitals (Herlev and North Zealand), including all patients operated in an emergency setting (*n* = 1116), including all patients with known cirrhosis at time of surgery. Postoperative complications, and mortality rates were evaluated by a matched case-control method, matching cases and controls according to surgical procedure, age, sex and American Society of Anaesthesiologists-class (ASA). Medical and surgical complications were classified according to the Clavien-Dindo classification.

**Results:**

In the study, 24 patients with cirrhosis and 48 matched controls were evaluated. The 30-day mortality was 37.5% for patients with cirrhosis and 12.5% for controls (OR 4.2, 95% CI [1.28, 13.80], *p* = 0.014) and 90-day mortality was 62.5% for patients with cirrhosis compared to 18.8% for controls (OR 7.22, 95% CI [2.41, 21.68], *p* < 0.001). For patients with cirrhosis 58.3% had surgical complications compared to 31.3% for the controls (*p* = 0.027). The reoperation rate was 45.8% in the cirrhosis group and 22.9% in the control group (*p* = 0.047). The days-alive-out-of-hospital at 90-days (DAOH-90) was 9 days in the cirrhosis group and 78 days in the control group (*p* < 0.001).

**Conclusion:**

This retrospective study shows that patients with cirrhosis have significantly higher mortality rates after emergency surgery, more surgical complications and reoperations, and reduced DAOH-90.

**Supplementary Information:**

The online version contains supplementary material available at 10.1007/s00068-025-02787-w.

## Introduction

Studies have shown an increased morbidity and mortality for patients with cirrhosis undergoing elective surgery, as well as a longer length stay at hospital, and more frequent admissions to intensive care units [1, 2]. The dysfunction of the cirrhotic liver affects all organs of the surgical patient with increased risks of infection, hemorrhage, and thrombosis. Anesthesia, stress response to the underlying trauma and the surgical trauma all aggregate the risk of hepatic decompensation and multi-organ-failure [3].

For emergency surgery on patients with liver cirrhosis, 30-day mortality rates vary from 19 to 40% [4, 5]. An eight-fold 30-day mortality increase was found after emergency surgery on patients with cirrhosis compared to elective surgery (40% compared to 5%) and twice the one-year mortality with 62% for emergent surgery on patients with cirrhosis compared to 33% for elective procedures [5]. The procedure specific data are sparse with studies reporting a mixture of patients undergoing different types of surgical procedures such as abdominal, orthopedic, cardiovascular, and neurosurgery [1, 2, 6, 7]. The aim of this study was to investigate the mortality and morbidity after emergency abdominal surgery in patients with liver cirrhosis, compared to a matched control group without cirrhosis, in a setting of state-of-the-art emergency perioperative care [9–11].

## Materials and methods

A retrospective cohort study was conducted from January 1st, 2019, to December 31st, 2019, at the Copenhagen University Hospital, Herlev (HEH), and from January 1st, 2021, to December 31st, 2023, at the Copenhagen University Hospital, North Zealand (NOH). The two hospitals serve a population of 755,000. All patients scheduled to emergency laparotomy or laparoscopy for ischemia, perforation of hollow viscus, bowel obstruction or intraabdominal hemorrhage were included. Patients below the age of 18 were not included in the database.

Data included patient demography, body-mass-index (BMI), American Society of Anaesthesiologists-class (ASA), WHO performance score, smoking, alcohol overconsumption, co-morbidities, medical records and data on the surgical procedure and the postoperative period, including postoperative complications registered according to the Clavien-Dindo Classification [8]. The following comorbidities were registered: diabetes mellitus, hypertension, atrial fibrillation, ischemic heart disease, and chronic obstructive pulmonary disease. In addition, cerebrovascular disease (previous stroke), dementia, chronic liver disease, chronic kidney disease, dialysis, cancer status (current local/disseminated, previous or none), ongoing oncological treatment (chemotherapy within 8 weeks), corticosteroids, immune modulating agents, and antithrombotic treatment were also registered.

All patients were treated according to a perioperative bundle care approach to emergency laparotomy with preoperative stabilization, early diagnosis, and surgery [9, 10], and a tailored intraoperative surgical strategy [11].

30-day postoperative complications were classified according to the modified Clavien-Dindo classification (CD) that grades any deviation or complication in the postoperative period according to severity and type of management [8]. CD I-II consists of any complications handled in the surgical ward, for example, correction of electrolytes, blood transfusion, treatment with antibiotics, superficial wound infections, etc. CD III-IV includes any complications demanding surgical-, radiological-, or endoscopic interventions and/or any complication requiring intensive care unit (ICU) management. Grade V is the death of a patient.

CD graded complications were divided into surgical complications and medical complications. Surgical complications included bleeding, abdominal wound dehiscence, bowel obstruction, wound infection, intra-abdominal abscess, anastomotic leakage, or other surgical complications, whereas medical complications were divided in cerebrovascular, pulmonary, cardiovascular, gastrointestinal, urogenital, and thromboembolic complications.

From this cohort of emergency surgery, patients registered with chronic liver disease were identified and included in this study. When the medical records were reviewed, patients with other chronic liver diseases than cirrhosis were excluded from the cirrhosis group.

Each patient with liver cirrhosis was matched manually to two controls (1:2) based on a propensity score which was estimated using logistic regression based on the specific surgical procedure, ASA-class, sex, and age. The medical records for the patients with cirrhosis were also examined for etiology of cirrhosis and confirmation of the diagnosis of cirrhosis as well as a noted Child-Pugh score [12].

Days alive and out of hospital at 90 days (DAOH-90) is a composite outcome that accounts for survival and time spent in hospital and it reflects a patient-centered approach by focusing on the patient’s quality of life and functional status outside of the hospital.

IBM SPSS version 29 (IBM Corp, NY) was used for statistical analysis. A p-value < 0.05 was considered statistically significant. Categorical data was summarized and presented as numbers and percentages of a group. Chi-square test was used for categorical data. For ASA scores in Table 1, Fisher’s exact test was used. To estimate the survival function, the Kaplan-Meier method was used. Survival time was defined as the date of surgery to the date of death. Censored data included only patients who had not died at the end of the follow-up (January 21st, 2025). Continuous data (age, BMI, length of hospital stay, and “days alive and out of hospital at 90 days”) were summarized and reported as medians with interquartile ranges and analyzed by Mann-Whitney U non-parametric test. GraphPad Prism version 10.4.1 for Windows (GraphPad Software, Boston, MA) was used to plot the Kaplan-Meier survival curve.

The primary outcomes were 30-day mortality and 90-day mortality. Secondary outcomes were frequency and severity of postoperative complications and days alive and out of hospital.

## Results

During the study period, 1116 patients underwent emergency surgery for perforation, bowel obstruction, ischemia, or intraabdominal hemorrhage. Of these, 31 were registered as having chronic liver disease. The medical records of these 31 patients were screened and 24 patients were identified with a diagnosis of liver cirrhosis. The seven patients not included: Four with alcoholic steatohepatitis, but not cirrhosis; one with portal hypertension of other etiology; one with primary biliary cholangitis (PBC) without cirrhosis; and one with viral hepatitis and alcohol abuse. These 24 patients with liver cirrhosis were then matched 2:1 with a control group.

Table 1 shows no significant statistical difference between the case and control group except for age, WHO performance score, alcohol intake, active cancer, and antithrombotic medication use. For 18 patients (75%) the etiology of cirrhosis was solely alcohol abuse, for two (8%) it was non-alcoholic steatohepatitis (NASH) with cirrhosis, one (4%) the cause was auto-immune hepatitis, one (4%) a combination of PBC and alcohol abuse, and one (4%) a combination of hepatitis C and alcohol abuse. For one patient (4%) no etiology was noted.

**Table 1:** Demographics

*n* = 72 (%)		Cirrhosis *n* = 24	Controls *n* = 48	*p*
Sex	Male	11 (45.8%)	23 (47.9%)	0.867
Age (median, IQR)		66 (56–72)	73 (64–78)	**0.016**
BMI (median, IQR)		25 (20–27)	24 (22–27)	0.984
ASA score*	1–2	0 (0%)	5 (10.4%)	0.162
	3+	24 (100%)	43 (89.6%)	
WHO Performance score	1–2	12 (50%)	39 (81.3%)	**0.023**
	3+	9 (37.5%)	8 (16.7%)	
	*(missing)*	*3 (12.5%)*	*1 (2%)*	
Alcohol overconsumption**		16 (66.7%)	17 (35.4%)	**0.018**
Smoking		12 (50%)	20 (41.7%)	0.448
*Previous medical history*				
Stroke		1 (4.2%)	7 (14.6%)	0.185
Dementia		2 (8.3%)	5 (10.4%)	0.778
Hypertension		12 (50%)	18 (37.5%)	0.310
Atrial fibrillation		2 (8.3%)	9 (18.8%)	0.247
Ischemic heart disease		5 (20.8%)	16 (33.3%)	0.271
Diabetes mellitus		3 (12.5%)	8 (16.7%)	0.643
COPD		4 (16.7%)	14 (29.2%)	0.248
Chronic Kidney Disease		4 (16.7%)	4 (8.3%)	0.289
Dialysis		0 (0%)	1 (2.1%)	0.498
Cancer*	None / former	24 (100%)	37 (77.1%)	**0.012**
	Local / disseminated	0 (0%)	11 (22.9%)	
*Medication*				
Chemotherapy < 8 weeks		0 (0%)	3 (6.3%)	0.211
Immunotherapy		2 (8.3%)	4 (8.3%)	1.000
Corticosteroids		2 (8.3%)	4 (8.3%)	1.000
Antithrombotics		4 (16.7%)	22 (45.8%)	**0.015**
*Liver disease*				
Child-Pugh class	A	4 (16.7%)	-	
	B	9 (37.5%)	-	
	C	4 (16.7%)	-	
	*missing*	7 (29.2%)	-	

COPD = Chronic obstructive pulmonary disease

*For statistical analysis, Fisher’s exact test was used, as none of the patients with cirrhosis could be classified as ASA 1–2; the same applies in the local/disseminated cancer group with cirrhosis

*Overconsumption was defined as “above maximal recommendation by National Board of Health”. In the study period equal to 7 units of alcohol/week (84 g) for females and 14 units of alcohol/week (168 g) for males

Table 2 shows the surgical procedures that were performed. The largest group was gastric- and duodenal ulcers (33.3%), followed by adhesiolysis (16.7%), small bowel resection (12.5%) and sigmoid colectomy (Hartmann’s procedure) (12.5%).

**Table 2:** Surgical procedures

*n* = 72	Cirrhosis *n* = 24	Controls *n* = 48
Perforated gastroduodenal ulcer	8 (33.3%)	16 (33.3%)
Adhesiolysis	4 (16.7%)	8 (16.7%)
Small bowel resection	3 (12.5%)	6 (12.5%)
Sigmoid colectomy (Hartmann)	3 (12.5%)	6 (12.5%)
Laparotomi and drainage	1 (4.2%)	2 (4.2%)
Laparoscopic drainage	1 (4.2%)	2 (4.2%)
Umbilical hernia repair	1 (4.2%)	2 (4.2%)
Ileocecal resection	1 (4.2%)	2 (4.2%)
Right hemicolectomy	1 (4.2%)	2 (4.2%)
Subtotal colectomy	1 (4.2%)	2 (4.2%)

*p* = 1.0 (intended, the patients are matched 1:2 based on operation type)

**Fig. 1:**
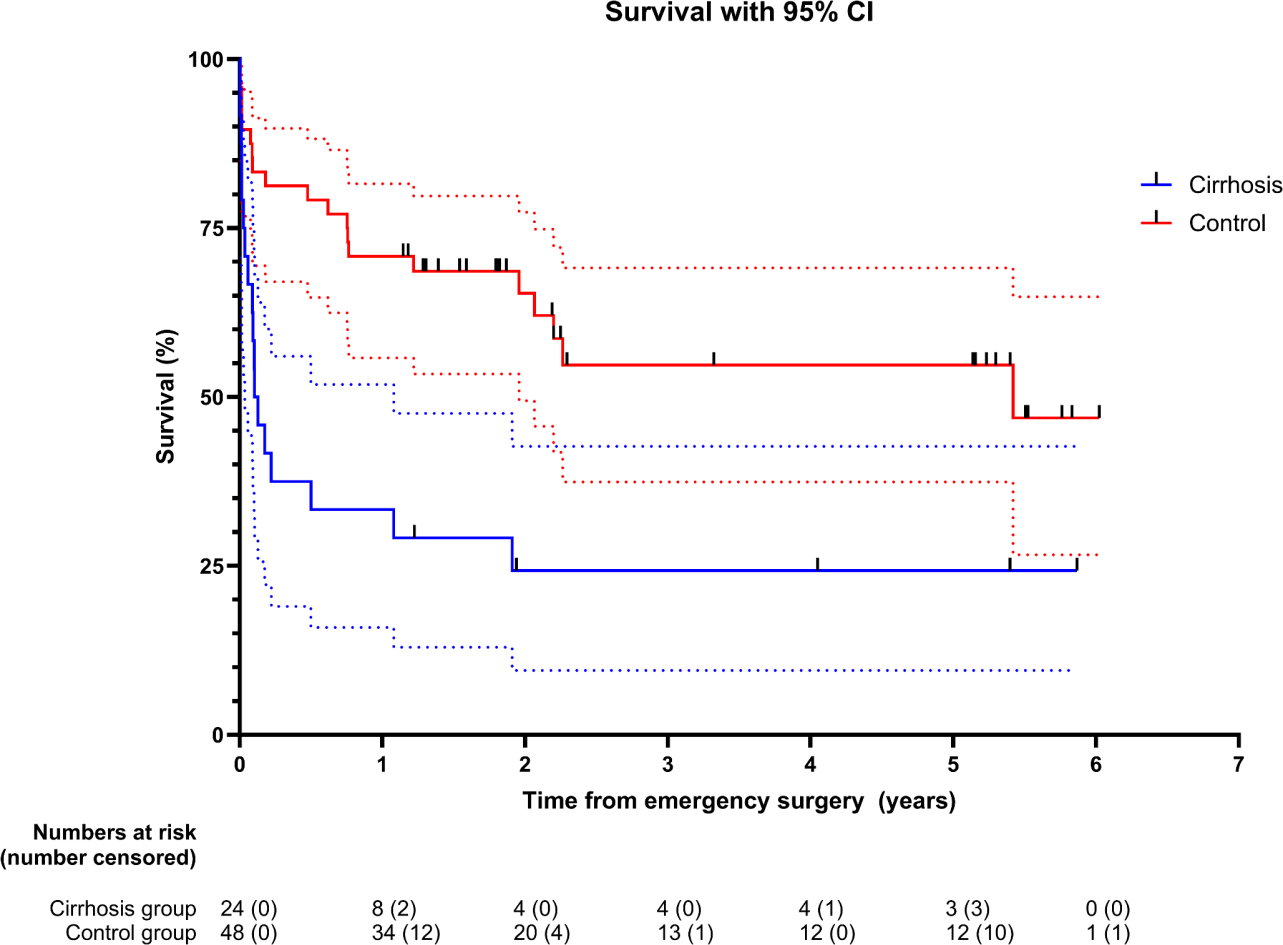
Kaplan-Meier plot of survival following emergency surgery

Table 3 shows the medical- and surgical complication rates, the length of stay, and the 30-day, 90-day and 1-year mortality rates. For the patients with cirrhosis 9 (37.5%) died within 30-days after surgery compared to 6 of the controls (12.5%), OR 4.20, 95% CI [1.28, 13.80], *p* = 0.014. At 90-days, the mortality rates were 15 of 24 (62.5%) and 9 of 48 (18.8%), respectively, OR 7.22, 95% CI [2.41, 21.68], *p* < 0.001. The one-year mortality was 16 of 24 (66.7%) for patients with cirrhosis and 13 of 48 for controls (27.1%), OR 5.39, 95% CI [1.86, 15.56], *p* = 0.001. Figure 1 shows a Kaplan-Meier survival plot for cirrhosis patients and controls. Median follow-up times were 43 days (IQR 12–572) in the cirrhosis group and 657 days (IQR 276–1544) in the non-cirrhosis group. The longest follow-up time was more than 6 years.

**Table 3:** Outcomes, mortality, and morbidity

*n* = 72 (%)	Cirrhosis *n* = 24	Controls *n* = 48	*p*
*Mortality*			
30-day mortality	9 / 24 (37.5%)	6 / 48 (12.5%)	**0.014**
90-day mortality	15 / 24 (62.5%)	9 / 48 (18.8%)	**< 0.001**
1-year mortality	16 / 24 (66.7%)	13 / 48 (27.1%)	**0.001**
*Complications*			
Length of hospital stay, median (IQR)	14 (6–32)	10 (6–18)	0.321
DAOH-90*, median (IQR)	9 (0–71)	78 (63–81)	**< 0.001**
Patients in ICU, n	18 (75%)	29 (60.4%)	0.370
Surgical complications, patients	14 (58.3%)	15 (31.3%)	**0.027**
Minor (CD 1–2)	7 (29.2%)	5 (10.4%)	**0.044**
Major (CD ≥ 3)	11 (45.8%)	13 (27.1%)	0.112
Reoperations**	11 (45.8%)	11 (22.9%)	**0.047**
*Surgical complications (events)*			
Bleeding	4	3	0.230
Ileus / bowel obstruction	2	0	0.128
Wound infection	6	6	0.369
Intraabdominal abscess	4	2	0.070
Anastomotic leak	4	1	0.064
Fascial dehiscence	5	3	0.065
Other	5	7	0.284
Medical complications, patients	21 (87.5%)	39 (81.3%)	0.502
Minor (CD 1–2)	20 (83.3%)	31 (64.6%)	0.099
Major (CD ≥ 3)	12 (50%)	14 (29.2%)	0.083
*Medical complications (events)*			
Cerebrovascular	9	9	0.186
Pulmonary	8	20	0.473
Cardiovascular	9	9	0.118
Gastrointestinal	10	21	0.866
Urogenital	8	14	0.697
Thromboembolic	4	9	0.688
Other	10	13	0.618

* Days Alive and Out of Hospital, 90 days

** All reoperations, excluding superficial abscess drainage

Most patients were admitted to an intensive care unit; 18 (75%) of cirrhosis patients and 29 (60.4%) in the matched control group, *p* = 0.370.

Both the cirrhosis group and controls had long hospital stays, and days-alive-and-out-of-hospital was calculated at 90-days (DAOH-90) (Table 3).

Mortality rates at 90-days based on Child-Pugh scores A, B, and C were 25%, 33%, and 100% (*p* = 0.006), respectively, albeit small numbers analyzed (see Table 1).

For the patients with cirrhosis 14 patients (58.3%) had a surgical complication (CD 1–5) compared to 15 (31.3%) for controls, *p* = 0.027. More patients with cirrhosis needed a reoperation (11 cirrhosis patients, 45.8%), than in the control group (11 controls, 22.9%), *p* = 0.047. Medical complications were common in both the cirrhosis group (21 patients, 87.5%), and the control group (39 patients, 81.3%), *p* = 0.502. Regarding major complications (CD ≥ 3), there was a higher rate of both surgical and medical complications in the cirrhosis group, but the results were not significant. Major surgical complications occurred in 11 (45.8%) with cirrhosis and in 13 (27.1%) in the control group, *p* = 0.112. Major medical complications occurred in 12 (50%) and 14 (29.2%) patients respectively, *p* = 0.083.

## Discussion

To our knowledge, the present study is the first to examine a highly vulnerable patient group, patients with cirrhosis of the liver, undergoing major emergency abdominal surgery in an advanced perioperative care bundle setting. The study revealed a 30-day mortality of 37.5% for patients with cirrhosis compared to 12.5% for matched controls; with an odds ratio of 4.2 for dying within 30 days, compared to the matched controls. This is in line with earlier studies that also show high mortality rates for patients with cirrhosis. A case-control study from 2004 found a 30-day mortality of 19% for patients with cirrhosis [4], undergoing a broad scope of general surgery, however this study also included elective and minor procedures such as biopsies and drainage of abscesses. A study from 2014 [5] showed a 30-day mortality of 20% and 90-day mortality of 30% for patients with cirrhosis and found both emergent and major surgeries to be risk factors for 30-day- and 90-day-mortality. The authors argue that cirrhosis patients experience delayed deaths and that the 90-day-mortality rate should be used in analysis of cirrhosis patients undergoing surgery. The MELD score [13] also was validated against a 3-month mortality rate for this reason. The observed 90-day-mortality in our study was 62.5% in cirrhosis and 18.8% in the control group. From 90-days to 1-year, the mortality rate levels off, with a 1-year-mortality in our study of 69.6% for the patients with cirrhosis compared to 28.2% for the controls, with the continued increase in mortality in the control group being carried by cancer deaths. After matching of controls, we found significant differences in age, alcohol use, and cancer prevalence. Alcohol abuse is associated with cirrhosis and therefore is unadjusted. The age is skewed in favor of the cirrhosis group, which was significantly younger, despite this, there were higher morbidity and mortality. The cancer prevalence was higher in the control group, and unadjusted in matching, and this likely accounts for the late deaths (between 90-days and 1-year) in the control group.

The highly significant difference in 90-day-mortality rate in our study could be explained by the combination of emergency presentation, high-risk major abdominal procedures, and other co-morbidities of the patients, but it is very likely related to the underlying liver cirrhosis. Although emergency abdominal surgery continues to carry a high mortality rate, our results show that especially emergency surgical intervention on patients with cirrhosis may be a high-risk procedure. In elective procedures, patients with cirrhosis show a lower mortality rate compared to emergency procedures [4–6], suggesting that elective procedures might be a way to reduce mortality rates. This idea finds support in another study, which reports low mortality rates for elective procedures in general [2]. However, when comparing outcomes for emergency and elective surgery universally, there are several possible biases in the elective patient population, since patients with comorbidities, such as cirrhosis, are often considered as being too fragile for elective surgery resulting in an underrepresentation and therefore making the adverse effects of emergency surgery appear larger. When taking this potential bias into account, it seems recommendable to schedule an elective procedure ahead of time to avoid a high-risk emergency procedure [5]. Naturally, this pro et con discussion is not possible for every patient – in our study, almost 30% of the patients were not diagnosed with cirrhosis before presenting as an emergency case.

We found a significant difference in surgical morbidity between the groups, showing that the patients with cirrhosis suffer from more complications than the controls. We failed to significantly show that patients with cirrhosis developed major postoperative complications, requiring intervention (Clavien-Dindo ≥ 3). This might to be due to a relatively low number of cases with cirrhosis, only 24 patients with cirrhosis out of 1116 in total, underpowering the study to show significant major complications.

Both groups had long median length-of-stay in hospital and a high rate of admission to ICU, reflecting the severity of illness. There was no significant difference between the two groups, however, the results should be interpreted with caution, as the high mortality rate is reflected as a short length-of-stay in hospital. The calculation of DAOH-90 has been validated in emergency surgery [14], and we found a significant difference between the groups, albeit the result should be viewed in light of the high mortality rates.

A study of hernia repairs showed that cirrhosis patients were more likely to undergo emergency surgery and also found a 7-fold increase in mortality in emergency hernia repair in the cirrhotic group [15], suggesting that elective repair may be better suited for these patients. This corroborates our findings of high mortality rates in cirrhosis patients undergoing emergency surgery and thus should be kept in mind, when assessing cirrhosis patients for elective surgery – the risk during the elective surgery should also be viewed in the light of an emergency presentation.

The number of complications observed in our study corresponds to previously published results [5, 16]. There are only few studies covering postoperative complications in patients with cirrhosis, and there is no greater consensus in which complications to include. The most included complications are emergency respiratory distress syndrome (ARDS), sepsis, renal failure, liver failure and coagulopathy, but the inclusion of surgical complications as well as the severity of the complications vary [2, 5, 17].

The strength of this study lies in the well-defined population of patients undergoing emergency surgery, high validity of data, and the accuracy in registration of postoperative complications within the Clavien-Dindo Classification. A biopsy confirming cirrhosis-diagnosis was not available for all patients. The main limitations lie in the low number of included patients with cirrhosis. The cases are matched with controls for the best fit available, but there is a higher prevalence of alcohol consumption in the cirrhotic group and more cancer and antithrombotic use in the control group. Despite adjusting for age, matching the relatively young age of the cirrhosis patients in the control group could not be achieved. The patients in the control group are older, which may contribute to underestimating the negative effect of cirrhosis on all outcomes. The study is retrospective and despite matching with a control group to make the groups more comparable, all sources of bias cannot be eliminated. Future studies should apply a prospective design and include a larger sample size.

## Conclusion

In conclusion, our retrospective cohort study found that the patients with cirrhosis undergoing emergency abdominal surgery have higher 30-day-, 90-day-, and 1-year-mortality rates, lower DAOH-90 and develop more surgical complications, with a high risk of reoperation, when compared to a matched control group. Future studies should elaborate on risk stratification, intensified optimization, and preventive measures in the perioperative setting.

## Electronic supplementary material

Below is the link to the electronic supplementary material.

Supplementary Material 1

## Data Availability

No datasets were generated or analysed during the current study.
