# Supplementary material for: A high rate of mortality in liver cirrhosis patients after emergency abdominal surgery
Source: Eur J Trauma Emerg Surg. 2025 Feb 21;51(1):117. doi: 10.1007/s00068-025-02787-w (PMC11845415; doi:10.1007/s00068-025-02787-w)
Supplement: Supplementary file 1 — Supplementary Material 1 [file 68_2025_2787_MOESM1_ESM.docx]

A high rate of mortality in liver cirrhosis patients after emergency abdominal surgery

Anders Peter Skovsen, MD ^1,2^

Thomas Korgaard Jensen, MD, PhD ^2^

Ismail Gögenur, Professor, MD, DMSc ^3^

Mai-Britt Tolstrup, MD, PhD ^1,2^

1. Copenhagen University Hospital North Zealand, Department of Surgery, Dyrehavevej 29, 3400 Hillerød, Denmark
2. Copenhagen University Hospital Herlev, Department of Surgery, Herlev Ringvej 75, 2730 Herlev, Denmark.

Zealand University Hospital, Dept. Surgery, Center for Surgical Science, Lykkebækvej 1, 4600 Koege, Denmark

Corresponding author:

Anders Peter Skovsen

Department of Surgery, Copenhagen University Hospital Hillerød

Dyrehavevej 29, 3400 Hillerød, Denmark.

+45 4829 2089

[anders.peter.skovsen@regionh.dk](mailto:anders.peter.skovsen@regionh.dk)

ORCID Ids:

Anders Peter Skovsen: 0000-0001-5782-8173

Thomas Korgaard Jensen: 0000-0001-9589-0955
Ismail Gögenur: 0000-0002-3753-268X

Mai-Britt Tolstrup: 0000-0003-3372-9677

# ABSTRACT

**Purpose**: In the elective setting, there are high mortality rates for patients with liver cirrhosis after surgery. Few studies focus on emergency surgery. This study investigates mortality and morbidity of patients with cirrhosis undergoing abdominal emergency surgery.

**Methods:** In a database established at two Copenhagen University Hospitals (Herlev and North Zealand), including all patients operated in an emergency setting (n = 1116), including all patients with known cirrhosis at time of surgery. Postoperative complications, and mortality rates were evaluated by a matched case-control method, matching cases and controls according to surgical procedure, age, sex and American Society of Anaesthesiologists-class (ASA). Medical and surgical complications were classified according to the Clavien-Dindo classification.

**Results**: In the study, 24 patients with cirrhosis and 48 matched controls were evaluated. The 30-day mortality was 37.5 % for patients with cirrhosis and 12.5% for controls (OR 4.2, 95% CI [1.28, 13.80], p = 0.014) and 90-day mortality was 62.5% for patients with cirrhosis compared to 18.8% for controls (OR 7.22, 95% CI [2.41, 21.68], p < 0.001). For patients with cirrhosis 58.3% had surgical complications compared to 31.3% for the controls (p = 0.027). The reoperation rate was 45.8% in the cirrhosis group and 22.9% in the control group (p = 0.047). The days-alive-out-of-hospital at 90-days (DAOH-90) was 9 days in the cirrhosis group and 78 days in the control group (p < 0.001).

**Conclusion**: This retrospective study shows that patients with cirrhosis have significantly higher mortality rates after emergency surgery, more surgical complications and reoperations, and reduced DAOH-90.

## **Keywords:**

## Liver cirrhosis, emergency surgery, mortality, morbidity, postoperative complications.

# Statements and Declarations

**Ethics approval and consent to participate**

Ethics committee approval number: 21000282 from hospital/regional board.

According to Danish law, individual patient consent is not required in this type of study.

All data were anonymously analyzed without individual patient consent.

**Consent for publication**

Not applicable

**Availability of data and materials**

All data generated or analysed during this study are included in this published article.

**Competing interests**

The authors declare that there are no financial or non-financial conflicts of interest.

**Funding**

This research received no specific grant from any funding agency in the public, commercial, or not-for-profit sectors.

**Authors' contributions**

All authors (APS, TKJ, IG and MT) contributed to the conception and design of the work.

APS and MT gathered data, performed statistical analyses and wrote manuscript. TKJ was a major contributor in writing the manuscript. IG performed substantial revisions.

All authors read and approved the final manuscript.

# Acknowledgements

The authors would like to thank Esben Holm Hansen, MD, and Sara Kehlet Watt, MD, PhD, for their contributions to the initial phases of the draft.
